# Identification of drought-tolerant and stable mung bean genotypes using stress tolerance indices

**DOI:** 10.1186/s12870-026-09706-0

**Published:** 2026-09-29

**Authors:** Mohamed R. Asaad, Hassan A. H. Soltan, Hanaa S. H. Bakry, Bahaa Abugammie, Islam M. Y. Abdellatif

**Affiliations:** 1 Department of Agronomy, Faculty of Agriculture, Minia University, El-Minia, 61517 Egypt; 2 Central Lab. of Organic Agriculture, Agricultural Research Center, Giza, Eg-12619 Egypt; 3 Department of Genetics, Faculty of Agriculture, Minia University, El-Minia, 61517 Egypt; 4 Department of Horticulture, Faculty of Agriculture, Minia University, El-Minia, 61517 Egypt

**Keywords:** Mung bean – drought tolerance, Stability Statistics, G×E interaction, AMMI – GGE-SCoT markers

## Abstract

**Background:**

Mung bean (*Vigna radiata* L. Wilczek) is an important grain legume increasingly cultivated in water-limited environments. However, strong genotype × environment interaction (GEI) often complicates the identification of broadly adapted and drought-tolerant genotypes. This study integrated multi-environment phenotypic evaluation with molecular characterization to identify superior mung bean genotypes for stressful environments.

**Results:**

Nine mung bean genotypes were evaluated under two irrigation regimes across two growing seasons. Environmental effects accounted for more than half of the total variation in seed and biological yields, while drought stress reduced seed yield by 25.66% across genotypes. Green seed fresh weight per pod, green pod fresh weight, and thousand-seed weight exhibited high broad-sense heritability coupled with high genetic advance, indicating favorable prospects for selection. Stress tolerance index (STI), geometric mean productivity (GMP), and mean productivity (MP) were strongly associated with grain yield under drought conditions. Multi-environment analyses revealed significant GEI, with the first two AMMI interaction principal components explaining 95.04% of the interaction variance. GGE biplot and complementary stability statistics consistently identified G8 as the highest-yielding genotype and G6 as the most stable across environments, whereas G8, G3, and G6 combined superior productivity with favorable yield stability. SCoT marker analysis generated 44 amplification products, of which 42 were polymorphic (95.45%), confirming substantial genetic diversity among the evaluated genotypes. The molecular analysis further supported the phenotypic differentiation of the superior genotypes, particularly the mutant-derived genotype G6, thereby strengthening confidence in its selection as a valuable breeding resource.

**Conclusion:**

The integration of drought tolerance indices, multi-environment stability analyses, and SCoT molecular markers provided a framework for identifying superior mung bean genotypes under contrasting irrigation conditions. G8 was identified as the most productive genotype, whereas G6 combined broad adaptation with high yield stability and detectable molecular differentiation from its parental genotype (G1). These findings provide valuable genetic resources for developing climate-resilient mung bean cultivars and demonstrate the advantage of combining phenotypic and molecular approaches to improve breeding decisions under water-limited environments.

**Supplementary Information:**

The online version contains supplementary material available at https://doi.org/10.1186/s12870-026-09706-0.

## Background

 Mung bean (*Vigna radiata* L. Wilczek) is an important grain legume cultivated widely due to its nutritional value, short growth duration, and adaptability to diverse agro-ecological conditions. It is grown across Asia, Africa, and other regions, where it contributes significantly to food security and sustainable agricultural systems [1]. The seeds are rich in easily digestible protein (22–28%), carbohydrates (60–65%), and essential micronutrients, including vitamins, minerals, and antioxidant compounds [2, 3]. In addition to its role as a staple food, mung bean is consumed in various forms such as dry seeds, sprouts, and processed products including noodles, pastes, and confectionery items, reflecting its wide culinary use [4, 5].

Beyond its nutritional importance, mung bean is valued in agricultural systems for its low input requirements and ability to fix atmospheric nitrogen, thereby improving soil fertility. Its relatively good tolerance to harsh environmental conditions further supports its cultivation under both temperate and tropical climates [6]. These attributes make it a promising crop for sustainable farming, particularly in regions facing increasing pressure from climate change and limited natural resources.

Despite these advantages, mung bean productivity remains constrained by several factors including environmental stresses and inconsistent yield performance across locations [7]. In comparison with major legumes, genetic improvement efforts in mung bean are still limited, and important gaps remain in understanding the extent of genetic variability for yield-related traits and stress tolerance [8]. In Egypt, mung bean has been introduced relatively recently as a summer crop and has shown potential in diversified cropping systems [9, 10]. However, its adoption is still restricted by limited awareness, weak value chains, and insufficient information on optimal agronomic practices such as planting density, sowing date, and irrigation management [9]. These constraints are further aggravated by increasing water scarcity and rising temperatures, highlighting the urgent need to identify mung bean genotypes capable of maintaining high and stable productivity under contrasting environmental conditions.

The key factor influencing crop performance under such conditions is the genotype-by- environment interaction (GEI), which describes the variation in genotypic responses across different environments. GEI arises because phenotypic expression is determined not only by genetic potential but also by environmental factors such as climate, soil conditions, and management practices. As a result, genotype (G) rankings may change across environments, making it difficult to identify consistently superior genotypes.

Understanding GEI is crucial for designing effective breeding strategies, particularly when selecting for traits such as yield stability under stress conditions. Reliable selection typically requires multi-environment trials conducted over different seasons and locations, and supported by appropriate statistical tools to separate genetic and environmental effects. Additionally, the additive main effects and multiplicative interaction (AMMI) model has gained considerable attention. This model combines analysis of variance with principal component analysis to provide a more detailed interpretation of GEI and allows clear graphical visualization of genotypic performance across environments [11]. Genotypes that exhibit both high yield and consistent performance are generally preferred, as they ensure stable production under varying conditions [12]. Similarly, the genotype main effect (G) plus-genotype-by-environment (GGE) biplot has become one of the most widely used graphical approaches for simultaneously evaluating genotype performance, stability, and mega-environment patterns [13].

Although multi-environment field evaluation remains the most reliable approach for identifying superior mung bean genotypes, phenotypic performance alone may not fully reflect the underlying genetic relationships because quantitative traits are strongly influenced by environmental conditions and GEI [14]. Therefore, integrating molecular characterization with agronomic evaluation provides complementary information for distinguishing genetically diverse genotypes, identifying promising parental materials, and supporting breeding decisions [15]. Among the available DNA marker systems, Start Codon Targeted (SCoT) markers are highly informative, reproducible, and gene-targeted markers that have been successfully used to assess genetic diversity in several legume crops [16, 17]. However, studies integrating SCoT marker analysis with multi-environment evaluation of mung bean genotypes remain scarce [17, 18].

Drought stress is one of the most critical abiotic factors limiting mung bean productivity worldwide. It negatively affects plant growth, photosynthesis, reproductive development, and ultimately yield [19]. The response to drought varies among genotypes, leading to strong GEI and complicating the identification of tolerant lines based on single-environment evaluations. Previous research has shown that genotypic performance and stability rankings can differ markedly between well-watered and water-limited conditions which emphasizes the importance of evaluating genotypes under both these environments [20]. This issue is particularly relevant in Egypt, where high temperature conditions and limited water resources cause significant challenges for summer crops.

Although numerous studies have investigated drought tolerance in mung bean, limited information is available regarding genotype stability across contrasting irrigation regimes under Egyptian conditions. Moreover, studies that integrate multi-environment stability analysis with molecular characterization to identify superior mung bean genotypes remain scarce. Therefore, these investigations were conducted to: (a) evaluate the stability and adaptability of nine mung bean genotypes, (b) analyze GEI using univariate and multivariate stability methods to identify genotypes with superior performance under normal and stressed irrigation conditions, (c) provide insights into breeding strategies that enhance drought tolerance and yield stability in mung bean, (d) select the best genotypes for their effective use in future breeding programs, and (e) characterize the genetic diversity and relationships among the evaluated mung bean genotypes using Start Codon Targeted (SCoT) markers and integrate molecular information with field performance to identify promising parental materials for future breeding programs.

## Materials and methods

### Plant material

Nine mung bean genotypes representing diverse genetic backgrounds were evaluated in this study (Table S1). The experimental material included one local variety (Kawmy 1 (G1)), obtained from the National Research Center, Egypt; four exotic breeder lines (KPS-2 (G2), VC6469-12-3-4 A (G3), VC6492-59 A (G4), and VC6510-151-1 (G5)) originating from Thailand and Taiwan; and four mutant lines (M.7 (G6), M.8 (G7), M.9 (G8), and M.11 (G9)), developed through induced mutagenesis at the Agronomy Department, Faculty of Agriculture, Cairo University, Egypt. These genotypes were used to assess the extent of genetic variation in growth traits, seed yield (SY) performance, stress response, and GEI across the tested environments.

### Experimental site and design

Field experiments were carried out during the summer growing seasons of 2024 and 2025 at the experimental farm of the Faculty of Agriculture, Minia University, Egypt (28°11′N, 30°73′E). In each season, two field trials were conducted simultaneously under two different irrigation regimes. Both trials were laid out in a randomized complete block design (RCBD) with three replications. Irrigation was applied using a furrow irrigation system. Soil moisture content was monitored throughout the growing season using a calibrated time-domain reflectometry (TDR) probe (FieldScout TDR 300, Spectrum Technologies, USA) at 3-day intervals and at two soil depths (0–15 and 15–30 cm) within the root zone. Field capacity (FC) was determined gravimetrically by saturating soil cores and allowing free drainage for 48 h, whereas the permanent wilting point was determined using a pressure plate apparatus at 1.5 MPa.

The non-stress treatment was maintained near field capacity throughout the growing season, while the drought-stress treatment was maintained at approximately 60% of field capacity. Irrigation timing was adjusted according to soil moisture measurements to maintain the target moisture level for each treatment. On average, irrigation was applied every 7–10 days under the non-stress treatment and every 12–15 days under the drought-stress treatment, depending on soil moisture depletion rates during each season.

Each experimental plot consisted of three rows, each 3 m long and spaced 60 cm apart, with plants spaced 20 cm within rows, resulting in a total plot area of 5.4 m².

The recommended agronomic and cultural practices for summer legume production in the El-Minia region were applied consistently across all treatments and seasons.

### Seasonal climatic description

The average minimum and maximum air temperatures, as well as relative humidity (%) data were obtained from Mallawy Agricultural Climate Station, El- Minia governorate, Egypt for the period from April to August for both experimental years. The average minimum temperature ranged from 8.7 to 19.43 °C in 2024, with a slight increase observed in 2025. The maximum temperature ranged from 36.69 to 46.43 °C in 2024, and from 41.86 to 49.97 °C in 2025 (Figure S1A). The average relative humidity ranged from 36.33% to 51.97% in 2024 and from 31.88% to 49.30% in 2025 (Figure S1B).

### Soil physical properties and irrigation management

Soil samples were collected from the 0–30 cm depth at the experimental site and analyzed for their physical properties at the Soil Science Laboratory, Faculty of Agriculture, Minia University. The results indicated that the soil had a clay loam texture, consisting of 54.5% clay, 36.0% silt, and 9.5% sand. The soil was slightly alkaline, with a pH of 7.9.

Soil moisture content at FC and permanent wilting point (WP) was determined gravimetrically for each trial and growing season. Available water (AW) was calculated as the difference between FC and WP (Table S2).

### Measured traits

Agronomic and yield-related traits were obtained at two growth stages to characterize both vegetative performance and productivity. To avoid border effects, all the observations were recorded from five randomly selected plants in the middle row of each plot for each genotype in each replication.

At 60 DAS, the following five traits were recorded: green pod fresh weight (GPFW, g), green pod dry weight (GPDW, g), green seed fresh weight per pod (GSFW, g pod⁻¹), green pod length (GPL, cm), and green pod diameter (GPD, mm).

At final harvest (100 DAS), the following eight traits were recorded: average growth rate (AGR, cm day⁻¹), seed yield per plant (SY, g plant⁻¹), number of branches per plant (B/P), number of pods per plant (P/P), number of seeds per pod (S/P), 1000 seeds weight (TSW, g), harvest index (HI, %), and biological yield per plant (BY, g plant⁻¹). HI % was calculated as the ratio of SY to BY.

### Drought tolerance indices

Stress tolerance indices for SY were calculated using the pooled mean values of non-stress (*Yp*) and drought stress (*Ys*) conditions across the two seasons to identify drought-tolerant genotypes among the nine mung bean genotypes evaluated. The indices included mean productivity (MP)= $\:\left({Y}_{p}\mathrm{+}{Y}_{s}\right)∕2$ [21], geometric mean productivity (GMP)= $\:\sqrt{{Y}_{p}\times\:{Y}_{s}}$ [22], stress susceptibility index (SSI)= [(1 – ($\:{Y}_{s}$/ $\:{Y}_{p}$) / (1 – ($\:\overline{{Y}_{s}}\mathrm{/}\overline{{Y}_{p}}$)] [23] and stress tolerance index (STI)= $\:{(Y}_{p}\times\:{Y}_{s\text{}}$) / $\:{\left(\overline{{Y}_{p}}\right)}^{2}$ [22].

$\:{Y}_{p}$and $\:{Y}_{s}$ represent the SY of a given genotype under non-stress and drought stress conditions, respectively, and Ȳp and Ȳs are the mean SY of all genotypes under non-stress and drought stress conditions, respectively.

To evaluate the linear relationships and biological associations among the investigated yield traits and the computed drought tolerance indices, Pearson’s correlation coefficients were calculated. Statistical significance was set at *P* < 0.05, *P* < 0.01, and *P* < 0.001. The resulting correlation matrix was visualized as a partitioned triangular heatmap using R software to illustrate the trade-offs between agronomic components and drought indices.

### Molecular analysis

Molecular analyses were conducted at the Molecular Genetics Laboratory, Department of Genetics, Faculty of Agriculture, Minia University, Egypt.

For DNA extraction, genomic DNA was extracted from 200 mg of young leaves samples collected from the nine tested genotypes following the method of [24]. DNA quality was checked on 1% agarose gel, and DNA concentration was determined using a Nano-spectrophotometer.

SCoT analysis, eight SCoT primers were used to assess the genetic diversity among the nine genotypes (Table S3). PCR amplification was performed in a 25 µL reaction containing 12.5 µL PCR Master Mix, 2.5 µL primer, 4 µL genomic DNA, and 6 µL distilled water. The PCR program consisted of an initial denaturation at 94 °C for 4 min, followed by 35 cycles of 94 °C for 50 s, annealing at 50 °C for 1 min, and 72 °C for 2 min, with a final extension at 72 °C for 7 min. PCR products were separated on 2% agarose gel stained with ethidium bromide and visualized under UV light. Fragment sizes were estimated using a 100 bp DNA ladder, and gel images were documented using a GBOX 230 V system. Bands were scored as present (1) or absent (0), and polymorphism percentages were calculated using GelAnalyzer V26.1. Cluster analysis based on the similarity matrix was performed using SPSS.

### Statistical analysis

#### Combined analysis of variance

A combined analysis of variance (ANOVA) was calculated following the procedure described by [25]. Genotype means for each trait were compared using Tukey’s HSD test at *P* ≤ 0.05. The standard error of the mean (SEm) and coefficient of variation (CV%) were also calculated.

Genotypic and phenotypic variance components were estimated from the combined ANOVA, and the following genetic parameters were computed for each trait: genotypic coefficient of variation (GCV%), phenotypic coefficient of variation (PCV%), broad-sense heritability ($\:{h}_{b}^{2}$), and genetic advance as percentage of the mean (GA%). The $\:{h}_{b}^{2}$ was estimated based on a combined analysis of variance across the environments. Therefore, it represents the proportion of total phenotypic variation attributable to genetic effects while accounting for environmental influences and GEI. Prior to estimating $\:{h}_{b}^{2}$, the environmental variance (σ²E) and GEI variance (σ²GEI) were separated from the total phenotypic variance (σ²P = σ²G + σ²GEI + σ²E + σ²error), following the method described by [26]. Consequently, the heritability estimates reflect the stability of genetic control across environments rather than genetic effects within a single environment. In addition to the $\:{h}_{b}^{2}$, the PCV and GCV were calculated as important genetic parameters to assess the magnitude of variability present among the evaluated germplasm [27]. Genetic advance was calculated assuming a selection intensity of 5% (i = 2.06), following the method of [28].

#### Stability analysis

Yield stability across the four environments was evaluated using both univariate and multivariate stability statistics. Univariate stability parameters included Wricke’s ecovalence ($\:{W}_{i}^{2}$), which quantifies the contribution of each genotype to the total GEI sum of squares [29]; the $\:{b}_{i}\:$and S²di proposed by [30], which describe genotypic response to environmental variation and deviation from linear response, respectively. The best measure of phenotypic stability is a genotype with a bi that is equal to zero, whose yields were almost the same across environments. However, in the presence of a significant bi of performance on environmental indices, either more (b > 1) or less (b < 1) than unity, it is proven that the genotype is responsive to favorable or unfavorable environmental conditions, respectively Kang’s yield-stability statistic (*YS*_*i*_), which simultaneously selects for yield and stability in a single index [31].

The non-parametric stability parameter was developed for the simultaneous selection of yield and stability based on [31] *YS*_*i*_. The *YS*_*i*_ stability component is based on [32] stability-variance statistic $\:{\sigma\:}_{i}^{2}$, which calculates a genotype’s contribution to the total GEI attributable to all genotypes in the test [31]. The genotypes with *YS*_*i*_ statistics exceeding the mean *YS*_*i*_ value are considered ideal for selecting those that combine high yield and stability.

Multivariate stability analysis was conducted using the AMMI model [33, 34], which partitions the total variation into additive genotype and environment main effects and multiplicative GEI components captured by successive interaction principal component axes (IPCAs). The AMMI stability index (ASI) was calculated following [35], while the weighted average of absolute scores (WAAS) was computed according to [36]. WAAS weights the IPCA scores by their proportion of explained GEI variance to produce a single composite measure of stability.

Genotypic adaptability and stability were further visualized using GGE biplot analysis [37], which integrates both genotype main effects and GEI into a single biplot constructed from the first two principal components of the genotype-centered and environment-standardized data matrix. GGE biplots were used to identify the “which-won-where” pattern, as well as to evaluate discriminating and representative environments and determine the ideal genotype based on simultaneous consideration of mean performance and stability.

Spearman’s rank correlation coefficients were calculated among all pairwise combinations of stability statistics and mean SY to assess the degree of agreement among methods and to identify the most informative stability measures for genotype ranking under the tested conditions.

Pearson’s correlation coefficients were calculated to assess the relationships among yield-related traits and drought tolerance indices. Statistical significance was evaluated at *P* < 0.05, *P* < 0.01, and *P* < 0.001. The resulting correlation matrix was visualized as a partitioned triangular heatmap using R software.

All combined analyses of variance, genetic parameter estimations, and Spearman’s rank correlation analyses were performed using R software version 4.3.2 [38]. Specifically, the agricolae package version 1.3-7 [39] was used for ANOVA and post hoc multiple-comparison procedures, while the ggcorrplot package version 0.1.4 [40] was used for correlation matrix visualization. Meanwhile, the AMMI and GGE biplot analyses, alongside the calculation of the Weighted Average of Absolute Scores (WAAS) and AMMI Stability Index (ASI), were conducted using the PBSTAT-GE version 3.0.3 online platform [41]; available at https://www.pbstat.com). Additionally, bar charts with error bars representing the standard deviation (SD) were generated using Microsoft Excel.

## Results

### Combined analysis of variance

Generally, ANOVA combined with multiple comparisons is used by breeders to compare the genotypic means across different environments [42]. In this study, the magnitudes of mean squares obtained from the combined analysis of variance, along with their significance levels for nine mung bean genotypes evaluated across four environments for green and dry pod yields and their component traits were calculated (Table 1). The results revealed highly significant differences (*P* < 0.01) among the genotypes for all studied traits, except for GPD and S/P.

**Table 1:** Combined analysis of variance and genetic parameters for yield and its associated traits under different watering regimes across four environments (two growing seasons × two irrigation regimes)

Source	df		GPFW (g)	GPDW (g)	GSFW (g pod⁻¹)	GPL (cm)	GPD (mm)	AGR (cm day⁻¹)	SY (g plant⁻¹)	B/*P*	*P*/*P*	S/*P*	TSW (g)	HI (%)	BY (g plant⁻¹)
Genotype	8	M.S	1.12**	0.06**	0.30**	2.95**	0.01^NS^	0.25*	66.49**	4.06*	830.12**	0.48 ^NS^	5.85**	138.70**	1255.11**
		EV%	54.29	30.76	40.05	39.34	20.41	29.19	25.61	24.26	38.26	11.41	57.39	3.07	21.51
Watering regimes	3	M.S	0.50*	0.02 ^NS^	0.09*	1.11*	0.01 ^NS^	0.08*	360.53**	9.42**	1211.64**	2.86**	2.15**	3038.30**	8570.14**
		EV%	9.01	3.35	4.51	5.55	7.41	3.25	52.08	21.11	20.94	25.36	7.91	25.23	55.07
G×WR	24	M.S	0.11*	0.01 ^NS^	0.03 ^NS^	0.48*	0.004*	0.11**	14.52**	1.71**	138.10*	0.53**	0.43 ^NS^	40.86**	300.57**
		EV%	15.78	18.07	12.30	19.34	30.24	39.47	16.78	30.68	19.09	37.79	12.51	2.71	15.45
Pooled Error	64	M.S	0.04	0.01	0.04	0.29	0.002	0.02	1.56	0.39	51.02	0.12	0.26	18.26	38.33
		EV%	17.35	40.21	37.45	31.25	33.59	21.10	4.81	18.79	18.81	22.03	20.77	3.23	5.25
Genetic Parameters															
GCV%			12.07	9.66	13.73	4.67	5.48	32.07	11.60	5.51	12.01	0.60	11.54	9.34	11.32
PCV%			13.74	11.28	14.17	5.58	10.51	62.41	16.52	10.17	15.09	3.68	12.47	13.57	16.56
$\:{\boldsymbol{h}}_{\boldsymbol{b}}^{2}$			77.14	73.30	93.86	70.03	27.14	26.40	49.33	29.31	63.41	2.63	85.69	47.38	46.75
GA (% of mean)			16.34	12.43	22.62	5.74	2.61	14.86	10.04	2.83	13.37	0.03	17.36	7.77	9.29

*M.S *Mean squares from combined analysis of variance, *EV% *percentage of explained variation, *GPFW *Green pod fresh weight (g), *GPDW *Green pod dry weight (g), *GSFW* Green seeds fresh weight per pod (g), *GPL *Green pod length (cm), *GPD *Green pod diameter (mm), *AGR *Average growth rate (cm day⁻¹), *SY *Seed yield plant⁻¹ (g), *B/P *Branches plant⁻¹, *P/P *Pods plant⁻¹, *S/P *Seeds pod⁻¹, *TSW *1000-seed weight (g), *HI *Harvest index (%), *BY *Biological yield (g plant⁻¹), *GCV *Genotypic coefficient of variations (%), *PCV *Phenotypic coefficient of variations (%),$\:{h}_{b}^{2}$ Broad sense heritability, *GA *Genetic advance

Statistically significant differences are indicated by *p ≤ 0.05 and **p ≤ 0.01; NS indicates non-significant differences

Environmental effects represented by the combination of watering regimes across two seasons and have significantly affected all studied traits except GPDW and GPD. The environmental effects caused 52.08% and 55.07% of the total variation (EV%) for SY and BY, respectively. This indicates that environmental influences played a greater role than genotypic differences in determining these traits, suggesting a high level of phenotypic plasticity. In contrast, GPFW and GSFW were more strongly influenced by genotypic effects, which explained 54.29% and 40.05% of the variance, respectively (Table 1).

The genotype by environmental interaction was significant for most of evaluated traits, indicating that genotypes responded differently under varying environmental conditions. However, this interaction was not significant for GPDW, GSFW, and TSW (Table 1), suggesting a relatively stable performance of these traits across different environments and a consistent genotypic response to environmental variation.

### Genetic parameters

The PCV and GCV are useful genetic parameters in detecting the amount of variability present in the germplasm [27]. Thus, these genetic parameters were recorded in this study to detect the variability among the nine-mung bean tested genotypes. The PCV was consistently higher than the GCV for all studied traits, reflecting the influence of environmental factors. However, the relatively small differences between PCV and GCV for traits such as GPFW (12.07 vs. 13.74), GSFW (13.73 vs. 14.17), GPL (4.67 vs. 5.58), and TSW (11.54 vs. 12.47) indicate that these traits are less affected by the environmental variations including soil moisture and climatic conditions (Table 1).

Furthermore, heritability together with high genetic advance serves as a valuable indicator for predicting the effectiveness of selection in identifying superior genotypes for yield and its related traits, while also helping to clarify both the genetic control of traits and the extent of environmental influence on their expression [27]. The $\:{h}_{b}^{2}$ was found high for GSFW (93.86%), TSW (85.69%), GPFW (77.14%), GPDW (73.30%), and GPL (70.03%); moderate for SY (49.33%), HI (47.38%), and BY (46.75%); and low for GPD (27.14%), AGR (26.40%), and S/P (2.63%). The GA% expressed as percentage of the mean, was highest for GSFW (22.62%), TSW (17.36%), and GPFW (16.34%), while it was very low for S/P (0.03%), B/P (2.83%), and GPD (2.61%). Moreover, GSFW, GPFW and TSW exhibited high heritability coupled with high genetic advance, indicating the predominance of additive gene effects and the potential effectiveness of selection for these traits. In contrast, GPD, B/P, and S/P showed low heritability along with negligible genetic advance, suggesting limited scope for improvement of these traits through direct selection (Table 1).

### Mean performance of genotypes at green pod stage

Plant breeders use the mean genotypic performance for evaluation of genotypes across various environments [43]. In this study, the mean performance of genotypes was recorded under normal and drought stress conditions. Although there were no marked differences in the phenotypic structure of green pods among the nine genotypes (Figure S_2_), but significant differences were observed in the related traits.

**Fig. 1:**
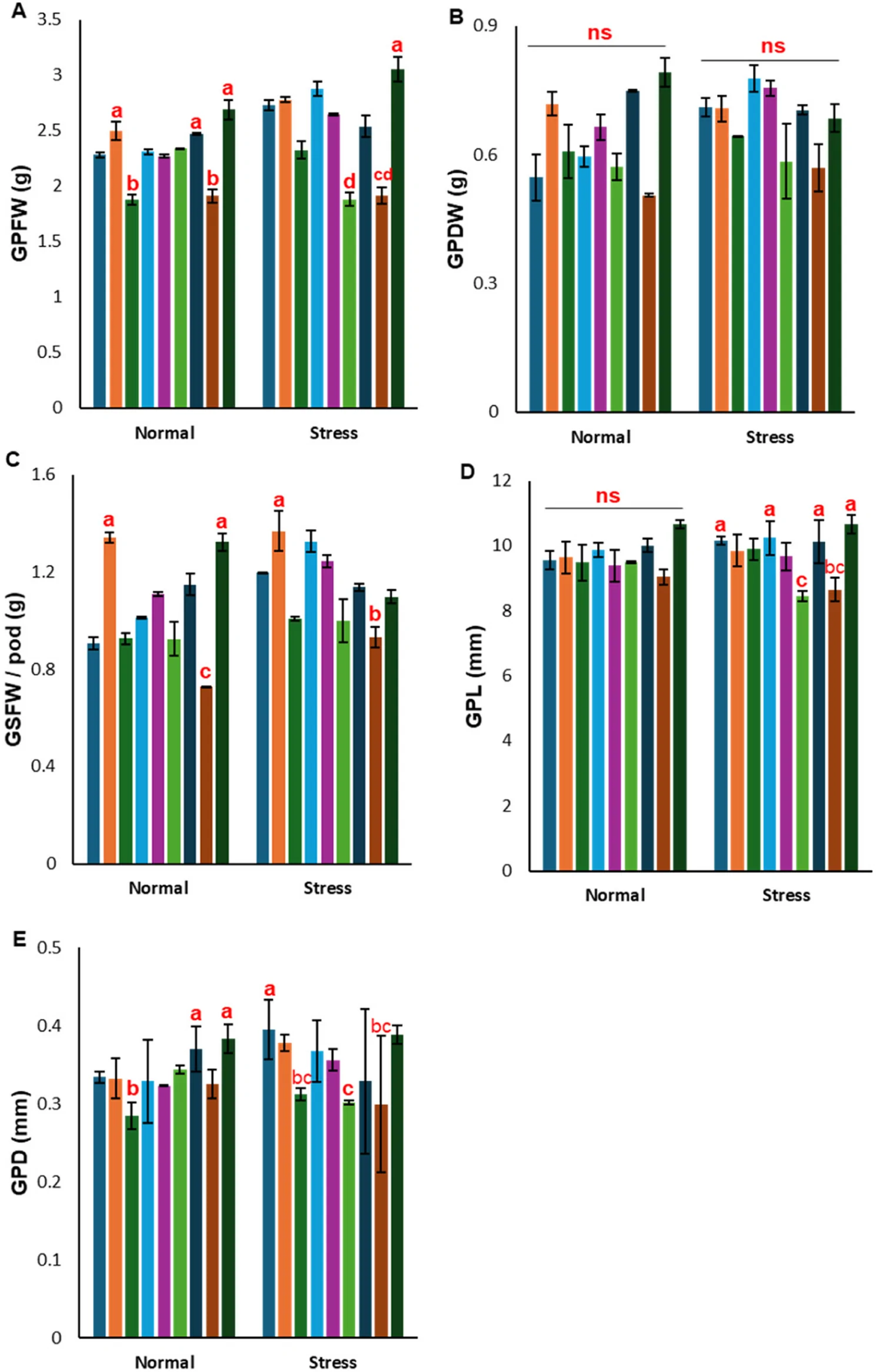
Mean performance of green pod-related traits of nine mung bean genotypes (G1–G9) under normal (non-stress) and drought stress conditions. **A** Green pod fresh weight (GPFW), (**B**) green pod dry weight (GPDW), (**C**) green seed fresh weight (GSFW), (**D**) green pod length (GPL), and (**E**) green pod diameter (GPD). Error bars represent the standard deviation (SD). Red letters indicate statistically significant differences; means sharing the same letter are not significantly different (ns). Units are grams (g) and millimeters (mm)

For the GPFW, the mean performance of genotypes under normal and stress conditions showed marked differences among them. Under normal conditions, the highest GPFW was recorded in G9 (2.69 g), followed by G2 and G7, while the lowest values were observed in G3 and G8. The remaining genotypes showed intermediate values and did not differ significantly from each other. Similarly, under stress conditions, G9 recorded the highest GPFW (3.05 g), whereas G6 showed the lowest value (1.88 g). Some genotypes, such as G4 and G2, maintained relatively high values under stress conditions. Thus, variation among genotypes was observed under both conditions, indicating different responses of genotypes to drought stress (Fig. 1A).

A similar pattern was found for GSFW per pod. Under non-stress conditions, G2 and G9 recorded the highest GSFW per pod (1.34 and 1.32 g, respectively), while G8 recorded the lowest value (0.73 g). Most of the remaining genotypes showed moderate values ranging from 0.90 to 1.15 g per pod. Under stress conditions, G2 remained the top-performing genotype (1.37 g), followed by G4 (1.33 g), whereas G8 produced the lowest value (0.93 g). In general, the differences among genotypes were less pronounced under stress, although G2 consistently performed well under both normal and stress conditions which indicates its better stability (Fig. 1B).

In contrast, GPDW did not differ significantly among the genotypes under either condition. Values ranged from 0.51 to 0.79 g under normal conditions and from 0.57 to 0.78 g under stress conditions, indicating limited genotypic influence and relative stability of this trait (Fig. 1C).

GPL showed non-significant differences among genotypes under normal conditions, with values ranging from 9.31 to 10.33 cm. However, under stress conditions, significant variation was observed. Genotype G9 recorded the highest pod length, followed by G4, G1, and G7, whereas G6 showed the lowest value. This suggests that pod length is more sensitive to stress conditions than under optimal growth environments (Fig. 1D).

For GPD, a moderate variation was observed among genotypes under both conditions. Under non-stress conditions, G9 and G7 recorded the highest values, while G3 showed the lowest. Under stress conditions, G1 recorded the highest diameter, whereas G6 showed the lowest values. Most genotypes were grouped in an intermediate statistical category, indicating relatively limited differentiation among them (Fig. 1E).

### Mean performance of genotypes at dry pod stage

Under normal conditions, G8 and G3 recorded the highest values of dry SY, while G4 and G7 showed the lowest values. Similarly, G8 remained the best-performing genotype under stress conditions, while G5 recorded the lowest SY (Table 2).

**Table 2:** Mean performance of yield-related traits of nine mung bean genotypes (G1-G9) under normal irrigation conditions and stress irrigation environments across two seasons

Under normal condition							
Genotype	SY (g plant⁻¹)	B/*P*	*P*/*P*	S/*P*	TSW (g)	HI (%)	BY (g plant⁻¹)
G1	18.95^bc^	9.37a	58.55b	11.09a	4.78b	29.74 cd	88.62bcd
G2	19.84^bc^	7.90ab	50.43c	10.55abcd	5.82ab	31.03bcd	85.37bcde
G3	25.23a	8.70ab	68.38a	10.65abcd	6.38a	34.54abc	99.97ab
G4	18.08c	7.87ab	51.00c	10.93abc	6.94a	28.20 cd	86.55bcde
G5	19.14bc	7.40b	59.74b	10.98abc	6.84a	25.77d	93.77abc
G6	22.30ab	8.77ab	65.35ab	10.43 cd	5.92ab	38.51ab	80.74cde
G7	17.09c	8.43ab	61.47ab	10.30d	5.74ab	30.16 cd	74.27de
G8	25.65a	8.57ab	68.21a	11.03ab	5.55ab	32.04abcd	109.30a
G9	19.59bc	8.50ab	47.11c	10.52bcd	6.58a	38.79a	70.10e
SEm	0.45	0.28	1.13	0.12	0.22	1.13	2.60
CV%	5.32	8.04	4.71	2.85	8.78	8.64	7.28

**Under stress environments**							
**Genotype**	**SY** (g plant⁻¹)	**B/P**	**P/P**	**S/P**	**TSW (g)**	**HI (%)**	**BY** (g plant⁻¹)
G1	14.95bcd	8.93a	40.83 cd	10.62a	4.07b	28.80ab	70.77bc
G2	12.75 cd	7.50abc	41.41 cd	10.30a	5.30ab	23.23b	68.58bc
G3	16.85ab	7.23bc	45.14bc	10.21a	6.11a	29.82ab	72.62b
G4	15.12bcd	7.57abc	43.31 cd	10.33a	6.47a	28.43ab	69.00bc
G5	12.45d	7.00bc	46.78bc	10.57a	5.89a	23.53b	69.46bc
G6	16.42abc	8.23abc	50.98ab	10.63a	5.64a	32.64a	68.30bc
G7	15.74abcd	7.63abc	42.84 cd	10.62a	5.36ab	32.42a	66.30bc
G8	18.91a	8.30ab	56.28a	10.83a	5.19ab	28.50ab	89.19a
G9	13.91bcd	6.73c	38.09d	10.33a	6.25a	33.98a	55.33c
SEm	0.56	0.23	0.95	0.15	0.20	1.64	2.45
CV%	9.08	7.48	5.15	3.55	8.87	13.82	8.57

Means followed by the same letter are not significantly different according to Tukey’s HSD test at *P* ≤ 0.05. The traits key names can be found in Table 1. The genotypes key names can be found in Table S1

*SEm *standard error of the mean, *CV*% coefficient of variation

For the number of branches per plant, G1 recorded the highest number under both normal and stress conditions, while the lowest values were observed in G5 under normal conditions and G9 under stress conditions. Most genotypes showed intermediate performance with a slight reduction after stress (Table 2).

The number of pods per plant also varied among genotypes. Under normal conditions, G3 and G8 had the highest pod numbers, while G9 recorded the lowest. This trend remained largely consistent under stress, with G8 maintaining the highest value and G9 the lowest. Furthermore, pod number among genotypes decreased under stress conditions (Table 2).

For the number of seeds per pod, there were significant differences among genotypes under normal conditions, where G1 showed the highest value, and G7 recorded the lowest value. However, the differences among genotypes were not significant under stress treatment (Table 2).

For TSW, G4, G5, G9, and G3 recorded the highest weight with nonsignificant differences among them, while G1 showed the lowest value under normal conditions. Under stress conditions, G4 and G9 remained among the highest, whereas G1 recorded the lowest value. A slight reduction in TSW was observed under stress conditions (Table 2).

Regarding harvest index, G9 and G6 recorded the highest values, while G5 showed the lowest value under normal conditions. Similarly, in addition to the G9 and G6 genotypes, G7 recorded the highest value of harvest index under stress, whereas G2 and G5 recorded the lowest values (Table 2).

For biological yield per plant, G8 consistently recorded the highest value under both conditions, while G9 showed the lowest value. As observed across all yield traits, biological yield decreased under drought stress in all genotypes, reflecting the cumulative effect of water deficit on vegetative growth, pod set, and seed filling processes (Table 2).

### Percentage reduction of yield-related traits

To quantify the magnitude of drought-induced yield loss across genotypes and traits, the percentage reduction under drought stress relative to normal irrigation was calculated for all major yield traits (Table 3). The mean percentage reduction in SY across all genotypes was 25.66%, while BY and P/P exhibited reductions of 19.89% and 23.09%, respectively. Among individual genotypes, G7 showed the smallest reduction in SY (7.90%), confirming its low stress susceptibility index (SSI = 0.30) and identifying it as the most drought-stable genotype in terms of yield maintenance. In contrast, G2 recorded the highest SY reduction (35.74%), consistent with its high SSI value (1.36) and indicating high sensitivity to water deficit. The relatively modest reduction in TSW across most genotypes (mean = 7.95%) compared with SY (mean = 25.66%) suggests that seed filling efficiency is partially buffered against moderate drought stress, whereas pod number per plant, which exhibited a mean reduction of 23.09% represents the primary yield component affected by water deficit in mung bean, consistent with established reports on legume responses to drought stress at the reproductive stage [19]. Notably, G6 recorded a negative reduction in HI (− 0.82%) and G7 in both S/P (− 3.11%) and HI (− 7.49%), indicating that these genotypes actually improved their harvest efficiency under stress conditions, possibly through reallocation of assimilates toward reproductive sinks when vegetative growth was constrained by water limitation.

**Table 3:** Percentage reduction of yield-related traits under drought stress relative to normal irrigation conditions

Genotype	SY (g plant⁻¹)	B/*P*	*P*/*P*	S/*P*	TSW (g)	HI (%)	BY (g plant⁻¹)
G1	21.11	4.70	30.26	4.24	14.85	3.16	20.14
G2	35.74	5.06	17.89	2.37	8.93	25.14	19.67
G3	33.21	16.90	33.99	4.13	4.23	13.67	27.36
G4	16.37	3.81	15.08	5.49	6.77	-0.82	20.28
G5	34.95	5.41	21.69	3.73	13.89	8.69	25.93
G6	26.37	6.16	21.99	-1.92	4.73	15.35	15.41
G7	7.90	9.49	30.31	-3.11	6.62	-7.49	10.73
G8	26.28	3.15	17.49	1.81	6.49	11.05	18.40
G9	28.99	20.82	19.15	1.81	5.02	12.40	21.07
Mean Reduction %	25.66	8.39	23.09	2.06	7.95	9.02	19.89

*The traits key names can be found in Table 1

### Drought-stress-tolerance indices

Stress tolerance indices were calculated using pooled mean SY values under non-stress (Yp) and stress (Ys) conditions across two seasons (Table 4). Clear differences in SY per plant were observed among the tested genotypes under both conditions during the two studied seasons. In the first season, Yp ranged from 17.09 g plant⁻¹ in G7 to 25.65 g plant⁻¹ in G8, whereas Ys varied from 12.45 g plant⁻¹ in G5 to 18.91 g plant⁻¹ in G8 (Table 4).

**Table 4:** Stress tolerance indices based on seed yield of nine mung bean genotypes (G1-G9) evaluated under normal and water-stress environments across two seasons

Genotypes	SY (g plant⁻¹)					
	$\:{Y}_{p}$	$\:{Y}_{s}$	STI	MP	GMP	SSI
G1	18.95	14.95	0.66	16.95	16.83	0.80
G2	19.84	12.75	0.59	16.30	15.90	1.36
G3	25.23	16.85	1.00	21.04	20.62	1.27
G4	18.08	15.12	0.64	16.60	16.53	0.62
G5	19.14	12.45	0.56	15.80	15.44	1.33
G6	22.30	16.42	0.86	19.36	19.14	1.00
G7	17.09	15.74	0.63	16.42	16.40	0.30
G8	25.65	18.91	1.14	22.28	22.02	1.00
G9	19.59	13.91	0.64	16.75	16.51	1.11

The genotypes key names can be found in Table S1

$Y_p$: Yield of mung bean genotypes under non-stress; $Y_s$: Yield of mung bean genotypes under drought stress

*STI *Stress Tolerance Index, *MP *Mean Productivity, *GMP *Geometric Mean productivity, *SSI *Stress Susceptibility Index

Based on the STI, GMP, and MP, G8 exhibited the highest values, followed by G3. This indicates their superior performances under both stress and non-stress conditions and suggesting good drought tolerance combined with high yield potential. In contrast, G5 recorded the lowest STI and GMP values, indicating its poor performance under drought conditions (Table 4).

Regarding SSI, G7 had the lowest value (0.30), indicating higher stability and low sensitivity to drought stress. On the other hand, G2 and G5 showed the highest SSI values (1.36 and 1.33, respectively) reflecting their greater susceptibility to drought and higher yield reduction under stress conditions (Table 4).

### Rank correlation

To elucidate the relationship between drought tolerance indices and SY under non-stress and stress conditions, Spearman’s rank correlation coefficients (* r*) was calculated (Table 5). The correlation between Yp and Ys was positive but non-significant (*r* = 0.47^NS^), suggesting that high yield potential under favorable conditions does not necessarily translate into high performance under drought stress (Table 5).

**Table 5:** Spearman’s rank correlation coefficients (r_s_) among seed yield and drought tolerance indices of nine mung bean genotypes (G1-G9) across two seasons

Ys	Yp	Ys	STI	MP	GMP
	0.47^NS^				
STI	0.63 ^NS^	0.87**			
MP	0.65 ^NS^	0.85**	0.99**		
GMP	0.60 ^NS^	0.88**	0.99**	0.98**	
SSI	0.55 ^NS^	-0.42 ^NS^	-0.23 ^NS^	-0.20 ^NS^	-0.27 ^NS^

Statistically significant differences are indicated by *p ≤ 0.05 and **p ≤ 0.01; NS indicates non-significant differences

Ys showed a strong and highly significant and positive correlation with STI (* r* = 0.87**), MP (* r* = 0.85**), and GMP (* r* = 0.88**). These results indicate that these indices are effective tools for identifying and ranking mung bean genotypes with better performance under drought conditions (Table 5). In contrast, the SSI showed non-significant correlations with all studied parameters, indicating its limited efficiency in discriminating genotypes based on their performance under stress in this study. The strong positive correlations among STI, MP, and GMP indicate a high level of agreement between these indices. Suggesting that they may be used interchangeably for selecting drought-tolerant genotypes in mung bean (Table 5).

### Correlation analysis between yield traits and drought tolerance indices

**Fig. 2:**
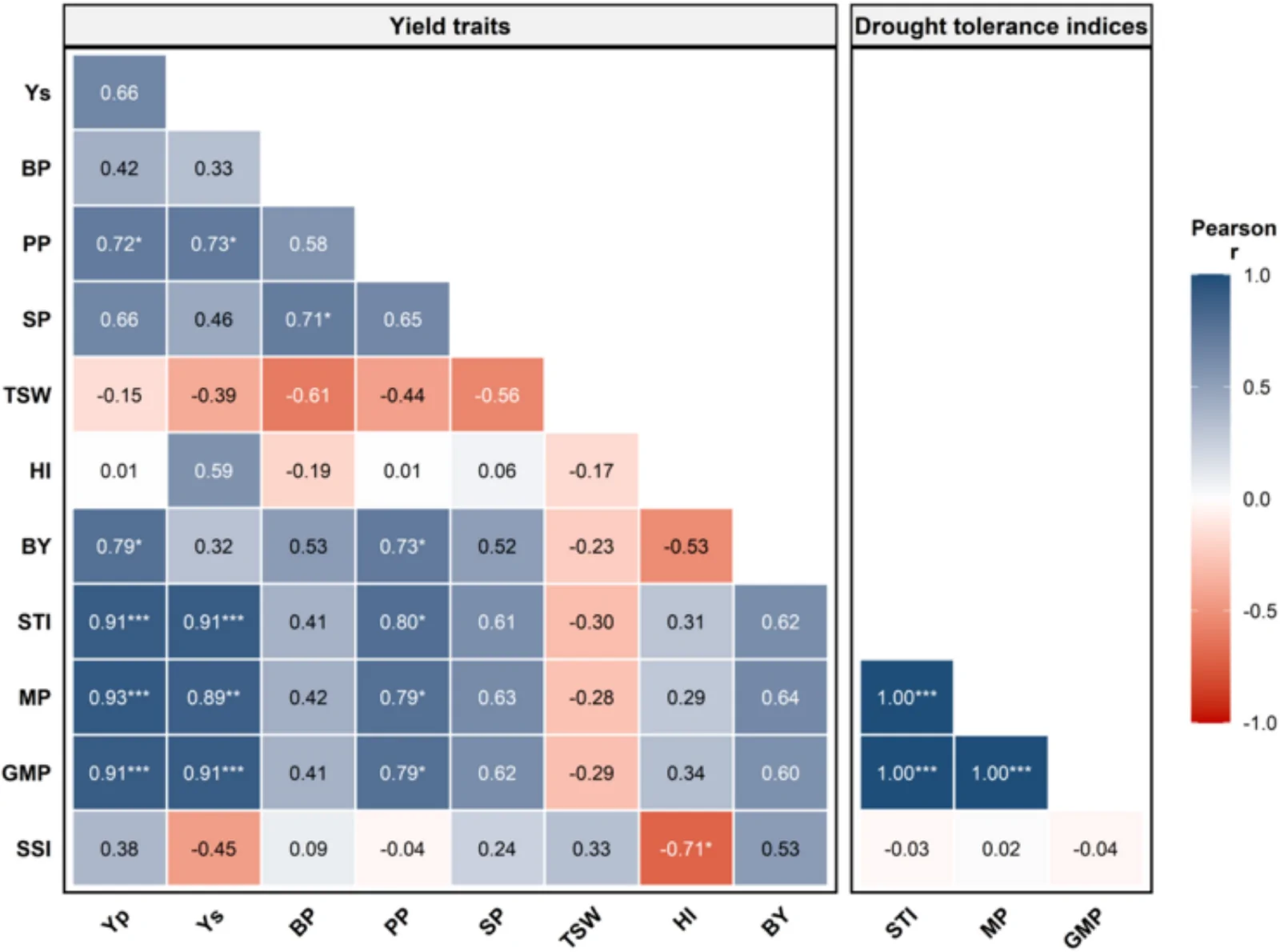
Pearson correlation heat map showing the relationships among seed yield under non-stress (Yp) and drought-stress (Ys) conditions, yield-related traits measured at harvest, and drought tolerance indices in nine mung bean genotypes evaluated across four environments. Correlation coefficients (r) are represented by color intensity, with blue indicating positive correlations and red indicating negative correlations. Asterisks denote significant correlations (*p ≤ 0.05, **p ≤ 0.01, ***p ≤ 0.001), whereas correlations without asterisks are not significant (*p* > 0.05). B/P = branches per plant; P/P = pods per plant; S/P = seeds per pod; TSW = thousand-seed weight (g); HI = harvest index (%); BY = biological yield per plant (g plant⁻¹); STI = stress tolerance index; MP = mean productivity; GMP = geometric mean productivity; SSI = stress susceptibility index

Pearson’s correlation analysis was performed to examine the relationships among seed yield under Yp and Ys conditions, yield-related traits, and the four drought tolerance indices (Fig. 2). The analysis revealed several meaningful associations among these variables. Yp showed strong positive correlations with STI, MP, and GMP (*p* ≤ 0.001), whereas its correlation with SSI was weak and non-significant. The relationship between Yp and Ys was positive but moderate (*r* = 0.66) and did not reach statistical significance, indicating that high yield under favorable conditions was not always associated with superior performance under drought stress.

Similarly, Ys was strongly and positively correlated with STI (*r* = 0.91***), MP (*r* = 0.89**), and GMP (*r* = 0.91***), while showing a moderate negative association with SSI (*r* = -0.45). These results indicate that STI, MP, and GMP were more effective than SSI in identifying genotypes capable of maintaining high productivity under drought stress conditions.

Among the yield-related traits, P/P exhibited significant positive correlations with both Yp (*r* = 0.72*) and Ys (*r* = 0.73*), as well as with STI (*r* = 0.80*) and MP (*r* = 0.79*). These relationships suggest that pod number is an important component contributing to yield performance under both normal and drought conditions. BY was also positively correlated with Yp (*r* = 0.79*) and PP (*r* = 0.73*), highlighting the contribution of biomass production to seed yield. In contrast, TSW showed moderate to strong negative correlations with several traits, including a negative correlation with branches per plant (BP; *r* = -0.61). TSW was only weakly associated with STI, MP, and GMP (*r* = -0.30 to -0.28), suggesting that seed size was not a major determinant of drought tolerance in the evaluated genotypes. HI displayed a significant negative correlation with SSI (*r* = -0.71*) and a moderate negative correlation with BY (*r* = -0.53).

The drought tolerance indices STI, MP, and GMP were very strongly and positively correlated with one another (*r* = 1.00***), indicating that they ranked genotypes in a highly similar manner. In contrast, SSI showed negligible correlations with these indices (*r* = − 0.03 to 0.02), suggesting that it captured a different aspect of genotypic response to drought stress (Fig. 2).

### Stability analysis

Performance and stability analyses of SY per plant among nine mung bean genotypes across four environments were conducted using univariate and multivariate stability statistics. Revealed that G8 produced the highest SY (22.28 g) across the tested environments, while G5 produced the lowest (15.79 g). Since the mean response was employed as the initial criterion for evaluating the genotypes under both non-stress and stress conditions in both growing seasons, 3 genotypes outperformed the grand mean (17.94 g) (Table 6).

**Table 6:** Mean performance and stability parameters of nine mung bean genotypes (G1-G9) across environments based on seed yield

Genotypes	Y_i_	$\:{W}_{i}^{2}$	$\:{b}_{i}$ ^1)^	$\:{S}_{di}^{2}$ ^2)^	ASI^3)^	WAAS^3)^	YS_i_^4)^
G1	16.95	9.12	0.60*	0.91 ^NS^	0.50	0.75	-4
G2	16.29	22.42	0.90 ^NS^	10.49**	0.97	1.06	-8
G3	21.04	10.63	1.33 ^NS^	2.60**	0.57	0.83	3+
G4	16.60	14.36	0.57*	2.95**	0.52	0.75	-6
G5	15.79	7.46	1.36*	0.59 ^NS^	0.46	0.68	-10
G6	19.36	1.16	1.05 ^NS^	0.004 ^NS^	0.17	0.23	9+
G7	16.41	25.12	0.63*	9.34**	0.94	1.30	-7
G8	22.28	23.22	1.54**	5.19**	0.87	1.25	4+
G9	16.75	2.65	1.00 ^NS^	0.80 ^NS^	0.24	0.29	3-
Mean	17.94						-1.8

The genotypes key names can be found in Table S1

*Y*_*i*_ Mean response,$\:{W}_{i}^{2}\:$Wricks’s (1962) ecovalance,$\:{b}_{i}$Regression coefficient (Finlay and Wilkinson 1963),$\:{b}_{i}\:$and$\:{S}_{di}^{2}$Deviation from regression (Eberhart and Russell 1966), *ASI *AMMI stability index (Jambhulkar et al. 2017), *WAAS *Weighted average of absolute scores (WAAS) (Olivoto et al. 2019) and *YS*_*i*_ yield-stability statistic (Kang 1993)

(1) * and ** indicate significant deviations of the regression coefficient () from unity (=1) at p ≤ 0.05 and р ≤ 0.01. (2) indicates stable genotype/s, whereas * and ** indicate unstable genotype/s at p ≤ 0.05 and р ≤ 0.01. (3) The lowest values for ASI or WAAS are the most desirable as stable. (4) + indicates stable and high-yielding genotypes selected on the basis of Kang’s yield-stability statistic (YSi)

In addition to $\:{W}_{i}^{2}$ parameter used for stability measures [12, 29], the $\:{S}_{di}^{2}$ parameter is also used for the same purpose and $\:{b}_{i}$ parameter for measuring the response. Under the tested environments, a significant difference was observed in most genotypes by $\:{b}_{i}$ and $\:{S}_{di}^{2}$. These results indicate that genotypes have responded differently to environmental changes (Table 6).

The genotypes, G1, G4 and G7 with $\:{b}_{i}\text{}$lower than one and close to unity, are moderate to low performer for SY and were adapted to drought conditions. The first ranked genotype in SY per plant performance was G8 with greater $\:{b}_{i}$ values than unity indicating high responsiveness to favorable environmental conditions and specific adaptation to non-stress environments (Table 6).

G6 demonstrated outstanding SY performance by the lowest $\:{S}_{di}^{2}\text{}$values and a substantial difference from zero. Conversely, G5, G9, and G1 exhibited the lower $\:{S}_{di}^{2}\text{}$values and moderate performance in SY. G6, G9, G5, and G1 were more stable because they recorded the minimum values of$\:\text{}{W}_{i}^{2}$ statistic. Furthermore, the minimum values of ASI and WAAS statistics indicate that the genotypes would be more stable across environments. G6 achieved a high SY and was the most stable genotype simultaneously because of their lowest values of ASI and WAAS compared to the other genotypes under investigated environments (Table 6). In this study, genotypes G8, G3, and G6 were identified as stable and high-yielding genotypes because their *YS*_*i*_ values were higher than the mean of *YS*_*i*_ -1.8.

### Rank correlation among mean yield and stability statistics

Spearman’s rank correlation coefficients for each pair of mean performance *Y*_*i*_ and stability statistics for SY were determined. The rank of genotypes based on mean SY showed a highly significant positive correlation with the *YS*_*i*_ index (*P* < 0.01), indicating strong agreement between yield performance and the combined yield–stability criterion (Table 7).

**Table 7:** Spearman’s rank correlation among seed yield and stability statistics of nine genotypes (G1–G9) evaluated across four environments

Statistics	rY_i_	$\:r{W}_{i}^{2}$	$\:{rb}_{i}$	$\:r{S}_{di}^{2}$	rASI	rWAAS
$\:{rW}_{i}^{2}$	0.07 ^NS^					
$\:r{b}_{i}$	-0.33 ^NS^	-0.15 ^NS^				
$\:r{S}_{di}^{2}$	0.13 ^NS^	0.93**	-0.27 ^NS^			
rASI	0.13 ^NS^	0.93**	-0.12 ^NS^	0.97**		
rWAAS	-0.03 ^NS^	0.95**	-0.03 ^NS^	0.88**	0.93**	
*rYS*_*i*_	0.91**	0.31 ^NS^	-0.30 ^NS^	0.33 ^NS^	0.38 ^NS^	0.25 ^NS^

Statistically significant differences at *p ≤ 0.05 and **p ≤ 0.01; ns: indicate the non-significant difference

The $\:{rW}_{i}^{2}$ showed strong and significant positive correlation with $\:r{S}_{di}^{2}$, rWAAS and rASI, suggesting a close relationship among these stability parameters in describing GEI effects. In particular, a highly significant positive association was observed between rASI and rWAAS (*P* < 0.01), indicating that both indices similarly rank genotypes in terms of stability across environments (Table 7).

In contrast, *bi* showed non-significant correlations with all other stability parameters, indicating that it represents a different aspect of genotype response compared with variance-based stability statistics. The strong positive correlation between *YS*_*i*_ and mean SY (*r* = 0.91**) confirms that this index effectively integrates both yield performance and stability, making it a reliable criterion for selecting superior genotypes. Therefore, the significant associations among W_i_², $\:r{S}_{di}^{2}$, ASI, and WAAS suggest that these stability measures are largely consistent in ranking genotype stability and therefore can be used interchangeably in breeding programs (Table 7).

### AMMI Analysis of variance

The AMMI analysis of variance for SY across the four environments revealed that the effects of environment (E), genotype, and GEI were highly significant (*P* < 0.01) (Table 8). This indicates substantial variation among environments, genotypes, and their interaction in determining SY performance.

**Table 8:** AMMI analysis of variance for seed yield per plant of nine mung bean genotypes (G1-G9) evaluated across four environments

S.O.V.	Df	Sum squares	Mean squares	F value	GEI variation explained (%)	
					Proportion	Accumulated
Environment (E)	3	1081.59	360.528	193.480**		
Replication/E	8	14.91	1.863	1.195 ^NS^		
Genotype (G)	8	531.92	66.490	4.580**		
G × E (GEI)	24	348.41	14.517	9.309**	58.81	58.81
PC1	10	204.91	20.491	13.140**	36.22	95.04
PC2	8	126.21	15.776	10.116**	4.96	100.00
PC3	6	17.29	2.881	1.847 ^NS^		
Residuals	64	99.80	1.559			

Statistically significant differences at *p ≤ 0.05 and **p ≤ 0.01; ns: indicate the non-significant difference

The AMMI model partitioned the GEI into principal component axes. The first interaction principal component axis (PC1) was highly significant (*P* < 0.01) and explained 58.81% of the GEI sum of squares. The second axis (PC2) was also highly significant and accounted for 36.22% of the GEI variation. Together, PC1 and PC2 accounted for 95.04% of the total GEI (Table 8), reflecting that most of the interaction pattern was effectively captured by the first two components.

In contrast, the third principal component axis (PC3) was not significant and contributed only 4.96% to the GEI variation (Table 8), suggesting that it had a minimal role in explaining this interaction.

### AMMI and GGE Biplot Analyses of GEI for SY

**Fig. 3:**
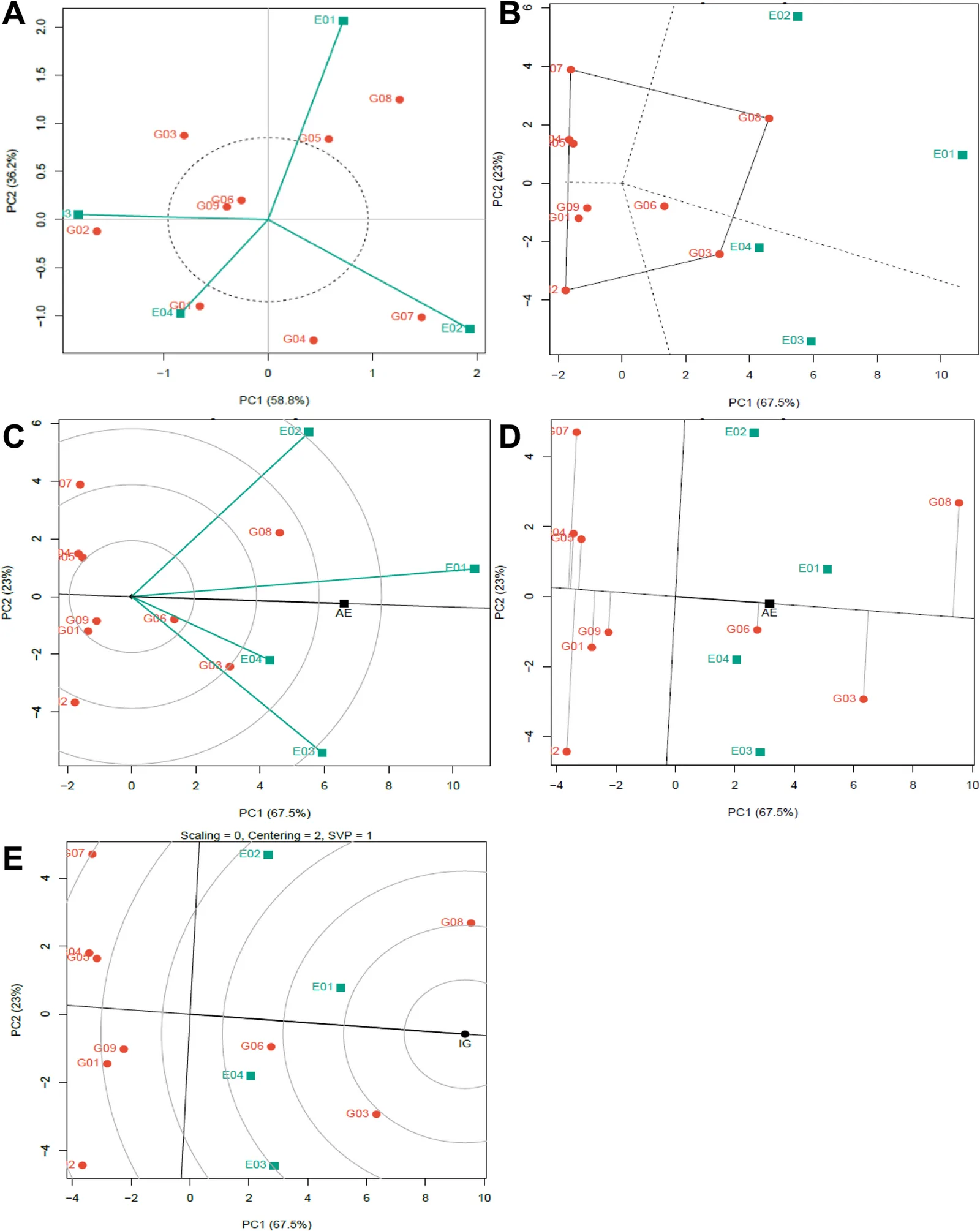
Multivariate analysis of seed yield performance and stability of nine mung bean genotypes across four environments. **A** AMMI biplot (PC1 vs. PC2) showing the interaction between genotypes (red) and environments (green). **B** GGE biplot polygon (“which-won-where”) illustrating the best-performing genotypes in specific environments. **C** GGE biplot of discriminativeness vs. representativeness of test environments. **D** GGE biplot of mean performance vs. stability of genotypes. **E** GGE biplot ranking of genotypes relative to the ideal genotype. Red symbols represent genotypes and green symbols represent environments

The AMMI biplot (Fig. 3A) provided a visual representation of the interaction patterns between the nine mung bean genotypes and the four tested environments. Among the environments, E1 produced the longest vector, indicating the highest contribution to GEI and the greatest discriminating ability among test environments. The vectors of E3 and E4 formed an acute angle, suggesting a positive correlation between these environments, whereas E2 showed a contrasting interaction pattern. Among the genotypes, G6 and G9 were positioned closest to the biplot origin, reflecting their relatively stable performance across all environments. In contrast, G4 and G7 were located far from the origin along PC1, indicating their strong contribution to GEI, while G8 showed a moderate distance from the biplot origin, suggesting intermediate interaction with the tested environments (Fig. 3A).

The GGE polygon biplot (Fig. 3B) identified G8, G7, G2, and G3 as the vertex genotypes, positioned farthest from the biplot origin in different directions, while the remaining genotypes fell within the interior of the polygon. The four environments were distributed across distinct sectors of the polygon. G8 produced the highest SY in the non-stress environments of both seasons (E1 and E3), while G7 achieved the highest SY under drought stress conditions in both seasons (E2 and E4). Genotypes G5, G4, G9, G6, and G1 fell within the interior of the polygon with no environments located within their corresponding sectors, indicating that none of these genotypes ranked as the highest-yielding entry in any of the four tested environments (Fig. 3B).

The discriminativeness versus representativeness view of the GGE biplot (Fig. 3C) was used to evaluate the capacity of each test environment to differentiate among genotypes and to represent the overall target environment. E1 possessed the longest environmental vector combined with the smallest angle relative to the average environment axis (AEA), identifying it as the most discriminating and representative test environment that is, the environment best suited for identifying superior genotypes with broad adaptability. E4 also demonstrated a relatively small angle with the AEA, ranking it as the second most representative environment. Strong positive correlations were observed between E1 and E4, as indicated by the acute angle between their vectors. In contrast, E2 and E3 showed wider angles with the AEA, reflecting lower representativeness of the overall target environment set (Fig. 3C).

The mean performance and stability biplot (Fig. 3D) classified genotypes according to their mean SY and stability simultaneously. G8, G3, and G6 were positioned to the right of the AEA ordinate, indicating above-average mean SY relative to the grand mean of 17.94 g plant⁻¹, while G7, G4, G5, G9, G1, and G2 were positioned to the left, indicating below average mean performance across all environments (Fig. 3D).

The ranking biplot relative to the ideal genotype (Fig. 3E) further clarified the relationship between productivity and stability among the nine genotypes. Among the above-average genotypes, G8 produced the highest mean SY across environments but exhibited a relatively longer projection from the AEA abscissa, indicating lower stability despite its superior mean productivity. G6 demonstrated the most favorable combination of high yield and stability among productive genotypes, as it was positioned closest to the AEA abscissa with a near zero projection, reflecting consistent performance across contrasting moisture environments. G3 also showed above-average yield but with lower stability than G6, as indicated by its longer vertical projection from the AEA abscissa. Among the below average genotypes, G2 and G7 showed the longest projections from the AEA abscissa, indicating the greatest instability within this group (Fig. 3E).

### Molecular characterization using SCoT markers

#### SCoT polymorphism

Genetic diversity among the nine-mung bean genotypes was assessed using eight Start Codon Targeted (SCoT) primers. All primers produced clear and reproducible amplification profiles (Figure S3), generating a total of 44 scorable DNA fragments, of which 42 were polymorphic, resulting in an overall polymorphism rate of 95.45% (Table 9). The number of amplified bands per primer ranged from 3 (SCoT-13) to 8 (SCoT-17), with fragment sizes varying between 226 and 2425 bp (Figure S3).

**Table 9:** Amplification profile of eight SCoT primers showing fragment sizes, polymorphic bands, unique bands, and percentage polymorphism across the studied mungbean genotypes

Primers	Fragment size (bp)	Monomorphic bands	Unique bands	Polymorphic bands		Total number of bands	Polymorphism (%)
				Without unique	With unique		
SCoT-2	387–2051	0	3	4	7	7	100
SCoT-13	269–663	1	0	2	2	3	66.67
SCoT-14	333–530	1	0	4	4	5	80
SCoT-17	441–2425	0	2	6	8	8	100
SCoT-18	430–2350	0	3	4	7	7	100
SCoT-19	226–1163	0	0	6	6	6	100
SCoT-20	327–992	0	0	4	4	4	100
SCoT-23	643–1369	0	0	4	4	4	100
Total		2	8	34	42	44	95.45

Among the eight primers, six (SCoT-2, SCoT-17, SCoT-18, SCoT-19, SCoT-20, and SCoT-23) exhibited 100% polymorphism, whereas SCoT-13 and SCoT-14 showed lower polymorphism levels of 66.67% and 80.00%, respectively, due to the presence of monomorphic fragments. In addition, eight genotype-specific (unique) bands were detected across the evaluated genotypes (Figure S3), indicating the high discriminatory capacity of the SCoT marker system.

#### Genetic similarity analysis

Genetic relationships among the nine mung bean genotypes were estimated using Dice’s similarity coefficient based on the polymorphic SCoT bands (Table S4). Similarity coefficients ranged from 0.195 to 0.800, indicating substantial molecular variation among the evaluated genotypes.

The highest genetic similarity (0.800) was observed between G4 and G6, followed by G3-G9 (0.792), G3-G7 (0.766), and G6-G9 (0.762), indicating close genetic relationships among these genotype pairs. In contrast, the lowest similarity coefficient (0.195) was recorded between G5 and G6, followed by G2-G6 (0.279) and G3-G5 (0.323), reflecting considerable genetic divergence.

The mutant genotypes (G6, G7, G8, and G9) exhibited moderate similarity with their parental genotype G1, with similarity coefficients ranging from 0.296 to 0.500. Nevertheless, appreciable molecular variation was also detected among the mutant genotypes themselves, with similarity coefficients (Table S4).

#### Cluster analysis

**Fig. 4:**
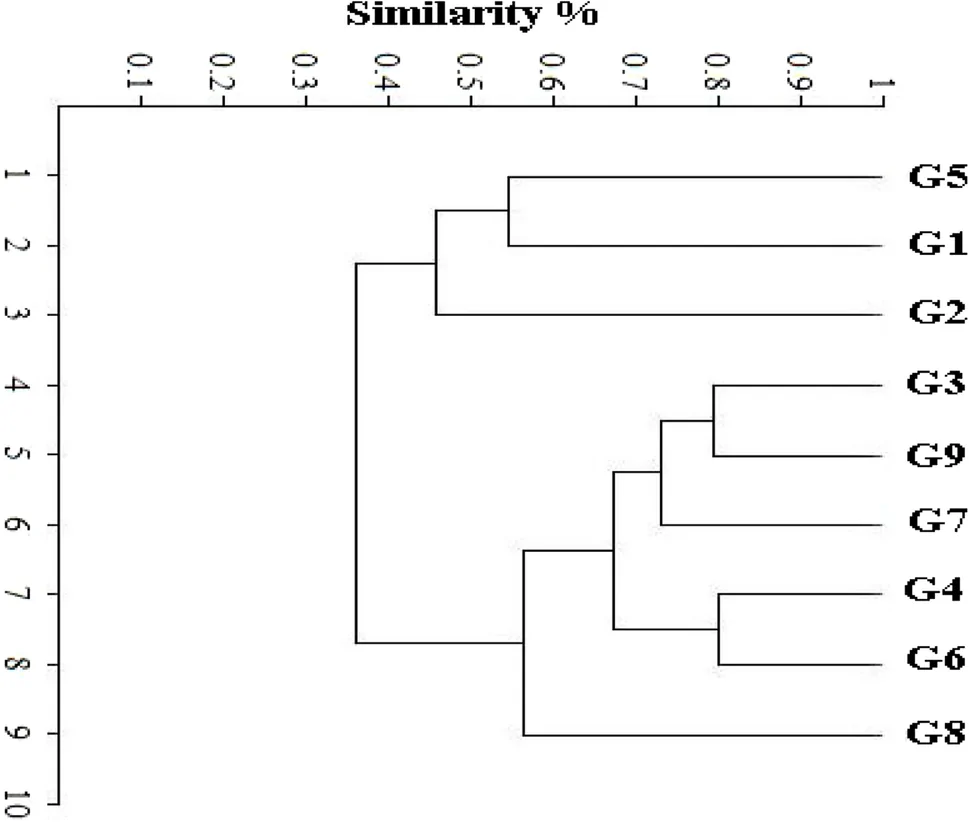
Dendrogram illustrating the genetic relationships among nine mungbean genotypes based on SCoT marker analysis

The dendrogram generated from the SCoT data classified the nine mung bean genotypes into two major clusters (Fig. 4). The first cluster comprised G5, G1, and G2, with G5 showing the closest relationship to G1. The second cluster contained the remaining six genotypes (G3, G4, G6, G7, G8, and G9) and was further subdivided into two sub-clusters.

One sub-cluster grouped G3, G7, and G9, whereas the second grouped G4 and G6, which also exhibited the highest similarity coefficient in the similarity matrix (0.800). Genotype G8 occupied a relatively distinct position within the second major cluster, indicating moderate divergence from the remaining members of this group.

The clustering pattern was generally consistent with the similarity coefficients estimated from Dice’s matrix, confirming the effectiveness of SCoT markers in resolving the genetic relationships among the evaluated mung bean genotypes.

## Discussion

Developing high-yielding cultivars with improved tolerance to abiotic and biotic stresses is a major objective of mung bean breeding. Among these stresses, drought remains a major constraint limiting mung bean productivity [44–46]. In this study, nine genetically diverse mung bean genotypes were evaluated under well-watered and drought-stressed conditions. Combined analysis of yield-related traits revealed significant differences in genotypic responses to irrigation regimes (Table 1), highlighting the importance of GEI in drought adaptation. Similar findings have been reported in previous multi-environment studies of mung bean [20, 47], indicating that reliable genotype selection requires evaluation across contrasting environments. The contrasting responses of the evaluated genotypes indicate substantial differences in their adaptation to water deficit which providing valuable information for identifying suitable parents for drought-tolerance breeding.

Genotype G2 exhibited the poorest performance under drought stress by recording the lowest SY together with high instability, as reflected by its unfavorable WAAS and YSi values (Table 6). The pronounced reductions in P/P and B/P suggest that yield loss was primarily associated with impaired branching and pod formation during the reproductive stage. Such reductions may result from increased competition for assimilates between vegetative and reproductive sinks under water deficit [19]. Furthermore, this response is consistent with the cellular dehydration and transcriptional disruptions that previously reported in drought-susceptible mung bean genotypes [48], indicating limited reproductive resilience under stress.

In contrast, Genotype G3 maintained relatively high yield potential (Table 2) but exhibited intermediate stability across environments (Table 6). Although it performed well under favorable conditions, its relatively large GEI indicates that its productivity was less predictable across contrasting irrigation environments. This pattern suggests that G3 is better suited to favorable target environments than to broad adaptation where stable performance across diverse conditions is required.

Genotype G7 exhibited the lowest SSI (Table 4) and the smallest reduction in SY (Table 2) under drought stress, suggesting superior drought tolerance. This response was accompanied by a negative reduction in HI (Table 3), indicating efficient assimilate partitioning toward seed production and reflecting an effective remobilization or drought escape strategy. However, despite its favorable performance under drought, stability statistics (high WAAS, low YSi, and a mean SY below the grand mean; Table 6) indicated that G7 performed inconsistently across environments. This suggests that G7 responded strongly to changes in environmental conditions rather than maintaining stable productivity over contrasting irrigation regimes. Such behavior is characteristic of phenotypic plasticity, whereby genotypes modify their performance in response to environmental variation instead of exhibiting broad adaptation [49].

Genotype G4 appeared to maintain seed-filling efficiency under drought stress, as indicated by the relatively small reduction in TSW (Table 3) despite a decline in pod number (Table 2). This response suggests that G4 preferentially maintained seed development under water deficit, a physiological buffering mechanism that has been associated with drought resilience in mung bean [48]. However, its relatively large contribution to GEI indicates that this adaptive response was not consistently expressed across all environments.

Genotype G8 combined superior yield performance with distinct environmental responsiveness. Similar to G4, G8 maintained relatively stable TSW despite reductions in pod number (Table 2), indicating efficient seed filling under drought conditions. Across all environments, G8 consistently recorded among the highest SY (Table 2), reflecting its strong yield potential. The GGE polygon biplot further identified G8 as the winning genotype under the non-stress environments (E1 and E3), demonstrating its excellent ability to exploit favorable growing conditions. The superior performance of G8 was associated with its higher P/P and greater SY (Table 2).

Overall, the AMMI and GGE analyses demonstrated that SY was strongly influenced by GEI, confirming that the evaluated genotypes responded differently to contrasting irrigation regimes. The GGE polygon biplot separated irrigated and drought-stressed environments into distinct sectors, illustrating a typical crossover GEI in which genotype rankings changed across environments [50]. Similar interaction patterns have been reported previously in mung bean [20, 51]. These findings indicate that favorable and water-limited environments impose different selection pressures on yield-related traits and therefore require different breeding strategies. The ability of the AMMI model to partition GEI and identify both high-yielding and stable genotypes is consistent with previous studies in mung bean [52–54] and highlights its value for parent selection under contrasting irrigation conditions [55].

Among the evaluated genotypes, G6 exhibited the greatest stability across environments. Its close proximity to the average environment axis in the GGE biplot, together with its favorable WAAS, ASI, and YSi values (Table 6), identified G6 as the most broadly adapted genotype under both well-watered and drought-stressed conditions. Although G6 showed a moderate reduction in SY under drought stress (Table 3), it maintained a consistently high HI and even exhibited an increase in S/P under stress (Table 2), suggesting efficient assimilate partitioning toward reproductive growth despite water limitation. Such conservative resource allocation has been associated with improved drought adaptation because maintaining reproductive development under stress contributes directly to yield stability [19, 56, 57]. Therefore, the consistent performance of G6 across contrasting irrigation environments indicates that it possesses valuable traits for broad adaptation. Nevertheless, the physiological mechanisms responsible for its superior stability require further investigation.

Molecular characterization using SCoT markers complemented the phenotypic evaluation by revealing substantial genetic diversity among the evaluated mung bean genotypes. The high polymorphism level (95.45%) demonstrates the ability of SCoT markers to discriminate closely related genotypes, which agrees with previous studies in mung bean and other legumes [17, 58, 59]. In addition, the clustering pattern was consistent with the genetic similarity matrix, confirming considerable molecular diversity within the evaluated germplasm.

Genotype G6 deserves particular attention. Although it was developed from the local cultivar Kawmy-1 (G1), it formed a separate cluster and showed only moderate genetic similarity to its parental genotype. This indicates that mutation breeding introduced detectable genomic variation while preserving much of the original genetic background. These molecular results agree well with the phenotypic evaluation, where G6 consistently showed superior stability across contrasting irrigation environments. Genotype G8 also occupied a relatively distinct position within the second major cluster and, at the same time, produced the highest SY, especially under well-watered conditions. Therefore, these findings suggest that G6 and G8 represent valuable genetic resources for future mung bean breeding.

The molecular and phenotypic results were largely consistent, increasing confidence in the identification of superior breeding materials. Whereas field evaluation identified genotypes with desirable agronomic performance, SCoT markers provided complementary information on their genetic diversity that was independent of environmental effects. Combining both approaches therefore offers a more reliable basis for parent selection than relying on either phenotypic or molecular evaluation alone.

Taken together, the integration of multi-environment phenotypic evaluation, AMMI and GGE analyses, stability statistics, and SCoT markers analysis highlighted G6, G8, and G3 as the most promising breeding materials for mung bean improvement under contrasting irrigation environments. Among these, G8 is recommended as a parent for improving yield potential under favorable irrigation, whereas G6 represents a valuable source of broad adaptation and yield stability across variable moisture conditions. The combined use of phenotypic and molecular approaches provides a more reliable basis for parent selection than either approach alone.

The environments evaluated in this study were generated from a single location by combining two growing seasons with two contrasting irrigation regimes. Although this design captured considerable environmental variation, further validation through multi-location trials across diverse agroecological regions is needed to confirm the stability and adaptability of the selected genotypes. Such evaluations will strengthen the applicability of the present findings and support the development of broadly adapted mung bean cultivars.

M.S: Mean squares from combined analysis of variance; EV%: percentage of explained variation; GPFW: Green pod fresh weight (g); GPDW: Green pod dry weight (g); GSFW: Green seeds fresh weight per pod (g); GPL: Green pod length (cm); GPD: Green pod diameter (mm); AGR: Average growth rate (cm day⁻¹); SY: Seed yield plant⁻¹ (g); B/P: Branches plant⁻¹; P/P: Pods plant⁻¹; S/P: Seeds pod⁻¹; TSW: 1000-seed weight (g); HI: Harvest index (%); BY: Biological yield (g plant⁻¹); GCV: Genotypic coefficient of variations (%); PCV: Phenotypic coefficient of variations (%); $\:{h}_{b}^{2}$: Broad sense heritability; GA: Genetic advance. Statistically significant differences are indicated by *p ≤ 0.05 and **p ≤ 0.01; NS indicates non-significant differences.

## Supplementary Information

Supplementary Material 1.

## Data Availability

All data are included in the published article and its supplementary materials.
